# The effects of psychopathology and shame on social representations of health and lifestyle behaviours via free association: a graph analysis approach

**DOI:** 10.1186/s40359-021-00671-x

**Published:** 2021-10-29

**Authors:** Evangelia Briseniou, Nikolaos Skenteris, Chryssi Hatzoglou, George Tsitsas, Epaminondas Diamantopoulos, Elena Dragioti, Mary Gouva

**Affiliations:** 1 Faculty of Medicine, School of Health Sciences, University of Thessaly, 41334 Larisa, Greece; 2 Research Laboratory Psychology of Patients, Families and Health Professionals, Department of Nursing, School of Health Sciences, University of Ioannina, 45500 Ioannina, Greece; 3 Ounseling Centre, Harokopio University, Athens, Greece; 4 Department of Health, Medicine and Caring Sciences, Pain and Rehabilitation Centre, Linköping University, Linköping, Sweden

**Keywords:** Social representations, Psychopathology, Shame, Free association, Centrality

## Abstract

**Background:**

There is a knowledge gap in whether psychopathology aspects can shape and mark the social representations about health and lifestyle. In this work, we investigated the association of psychopathology and shame with the centrality of the words describing eight common social representations of health and lifestyle.

**Methods:**

A convenience sample of 288 adults participated with an average age of 44.7, and 62.6% were women. The participants were asked to express three consecutive words associated with eight different health and lifestyle experiences by utilizing the free association method. The participants also were completed the Symptom Checklist-90-Revised (SCL-90-R), the Experiences of Shame Scale (ESS), and the Other as Shamer Scale (OAS). Canonical correlation analysis was applied to investigate the relationship between the set of the eight-word centralities and the psycho-demographic variables consisting of the subject's age and gender, the SCL 90 subscales, the OAS, and the ESS. Based on these findings, a structural equation explorative model was formed to test the unidimensionality of the five centralities construct.

**Results:**

Τhe psychological characteristics of interpersonal sensitivity, depression, external shame, and hostility were found to affect the word selection process on the social representations concerning nightlife, health, diet, lifestyle, and alcohol consumption. Participants with increased levels of depression tend to choose more centrally positioned words when the stimulus word was diet and more decentralized responses when the stimulus word was health. At the same time, higher external shame corresponded to more decentralized words for the categories of health and lifestyle.

**Conclusions:**

Our results indicate that there is a potential interaction between the psychological state and how a social representation of health and lifestyle is constructed through selected words. Graph theory emerged as an additional tool to use to study these relations.

**Supplementary Information:**

The online version contains supplementary material available at 10.1186/s40359-021-00671-x.

## Background

Social representations are a "theory of social knowledge" [1, 2] and play a key role in understanding and exploring health and illness behaviours [3–6]. It has been proposed that social representations are prescriptive of behavior and practices [3], which in turn may orient health and lifestyle choices and treatment preferences related to health and illness experiences [7]. According to Herzlich (1973), health and illness behaviours are those needing explanation, and they are attributed to the environment in which we live, the frenetic pace of life, an "unnatural" and unhealthy food, and air pollution [4]. Since then, the research on social representations of health and illness behaviours generally focuses on the multiplicity of beliefs of those behaviours and examines how these concepts are socially constructed and negotiated through the words in different communities [3, 5, 7, 8]. Furthermore, considering the significant health challenges of the twenty-first century, e.g., obesity, food, alcohol consumption, etc., the study of social representations may provide a valuable orientation to understand levels of knowledge about such health challenges [5].

The organization of social representations is formed by a dual structure; a central core and the peripheral elements [9]. The central core, which is quite stable, is purely social, linked to historical, sociological, and ideological assumptions, signifying the importance of the representation. The peripheral elements, which are more flexible, are determined more individually based on the experience of everyday life and allow adjustment to reality enabling “content differentiation” [9]. This dual structure has been widely used in the literature because it has a purposeful and evolution role in shaping social representations [3, 10–12].

Culture is one of the most established indicators of shaping and marking social representations within any community [3, 13, 14], through which people perceive and interpret their world [15]. For example, social representations about food, diet, wine, and health have strong links to cultural influences and cultural variations [3, 13, 14, 16]. However, the research of the structure of social representations concerning the role of other factors in shaping them within the same culture is limited, even though the social representations are made up of various components shared within the same community [17]. Individuals within a community are spoken objects with a certain cognitive and psychological state. This certain state includes a wide array of self-conscious experiences such as perception, beliefs, motivations, and positive or negative emotions including joy, happiness, sadness, fear, stress, anxiety, depression, and shame. The intensity and expression of a health and illness experience may depend on the dynamic patterns of such factors including one's culture permitting also psychological processes to be entailed in social representations. For example, the social representations of HIV/AIDS were greatly burdened with feelings of fear, shame, and sadness [18, 19]. Exploring and understanding the social representations about health and illness behaviours may, hence, require focusing on the reflections of psychopathology expressed through words in people within the same culture. Research findings showed that people with depression and anxiety provided a higher frequency of absolutist word usage and/or extreme response categories [20, 21]. At the same time, significant differences in the word used by depressed users were also reported in a study concerning Reddit users [22].

This study aims to contribute to highlight the relationship between psychopathological aspects and the structure of social representations by examining the effects of psychopathological aspects and shame on the words that describe eight common social representations of health and lifestyle behaviors, in the general population. We hypothesized that the resulting social representations would be varied in terms of central and peripheral structure concerning psychopathological differences within the same cultural context. We applied a novel analysis approach, where the words that were collected by using free association are represented in a network graph. At the same time, we calculated their centrality. Free word association evolved during four historical periods of Freud's studies and at present forms the primary tool of the projective techniques [23]. This method is considered to be a therapeutic process by psychoanalysis aiming to allow the subject to expose and describe its internal tensions [24, 25]. The free word association task has been previously exploited to provide insight into how the contents of social representations are constructed through words [3, 14].

Such a process is not shown at a group level since the inner network of meaningful connections for each individual is formed through its strictly personal and emotional interaction with the surrounding environment, thus acquiring a unique identity. However, within the same community, it is expected that common words will be created to express significant social representations related to life experiences common to all community members. Furthermore, the quantification of each word's graph position allows the participation of word data into further analytical procedures to identify statistically significant relations with the subject's psychopathological aspects as they were reflected by validated self-reported instruments.

## Method

### Participants and procedures

The data collection for this cross-sectional study was carried out in the spring and summer of 2019. A convenience sample of 294 Greek adults (i.e., ≥ 18 years old) using a snowball sampling method participated in the study. Details for study design and sampling methods have been described elsewhere [26]. In total, 110 men (37.4%) and 184 women (62.6%) aged 19 to 85, (M = 44.7, SD = 12.8) participated. The majority was married (N = 169, 57.5%) and university graduates (N = 198, 67.3%).

Written informed consent was obtained from all subjects, and all methods were carried out in accordance with the Declaration of Helsinki. The University of Thessaly (Larisa, Greece) local Ethics Committee approved the present study.

### Measurements

Participants completed a sociodemographic questionnaire, while they were asked to tell three first words that come to mind for eight common health and lifestyle behaviours, i.e., diet, exercise, smoking, alcoholic beverage, nightlife, lifestyle, disease, and health using the method of free association task (see Additional file 1: Free association questionnaire). These specific inductor words were selected based on the literature following Joffe's [5] notion of unhealthy lifestyle behaviours, i.e., dietary habits, physical inactivity, smoking, and alcohol.

Further, the Symptom Check List 90 (SCL-90-R), the Experiences of Shame Scale (ESS), and The Other as Shamer Scale (OAS) were given to the participants to evaluate certain types of shame experiences.

The Symptom Check List 90 (SCL-90-R) [27] is a widely used screening tool for assessing symptoms of psychopathology. It contains 90 items with a 5-point scale (0 = not at all, 4 = extremely), and assesses symptomatology in nine areas (Somatization; SM, Obsessive–Compulsive; OC, Interpersonal Sensitivity; IS, Depression; DEP, Anxiety; ANX, Aggression; AG, Phobia; PH, Paranoid Ideation; PI, Psychoticism; PS). The average score of all 90 items yields the global severity index (GSI), representing the overall level of distress and suggested to be the best single indicator of the current level of the disorder [27]. Higher scores on the scales of the SCL-90-R indicate higher distress; however, it should be noted that individual scales cannot be interpreted in diagnostic categories. The SCL-90-R had been translated into Greek [28]. The Cronbach a in our sample was 0.89.

The Experiences of Shame Scale (ESS) is a 25-item measure that assesses the frequency of shame experiences related to one's character ("Have you ever felt ashamed of the sort of person you are?"), behavior ("Have you tried to cover up or conceal things you felt ashamed of having done?"), and body ("Have you avoided looking at yourself in the mirror?"). Using a scale from 1 (not at all) to 4 (very much), participants rated the frequency of their shame experiences over the past year. Research has shown the ESS to have good discriminant and construct validity and high test–retest reliability [29]. The Cronbach alpha in our sample was 0.84.

The Other as Shamer Scale (OAS) is a modification of a subset of the items from the Internalized Shame Scale [30]. The original statements were rewritten to reflect a person's perception of what others feel about him or her, e.g. "I think that other people look down on me". The full scale consists of 18 items, inferiority (7 items), emptiness (4 items), how others behave when they see me make mistakes (6 items), while an item included in the total scale is not an item on any of the subscales. Answers range on a 5-point scale: 0 = “never,” 1 = “seldom,” 2 = “sometimes,” 3 = “often,” 4 = “almost always”. The Greek versions of the OAS and ESS questionnaires have been translated and validated into Greek by members of the research team [31, 32]. The Cronbach alpha in our sample was 0.85.

### Data preparation

Each word was typed separately in the datasheet. Words with a small conceptual difference and large frequency differences were grouped with a hyphen between them (e.g., the words "Διασκέδαση" and "Ψυχαγωγία" meaning "Fun/Entertainment" in Modern Greek formulate the compound word "Fun – Entertainment" since it was considered that they express a similar underlying perception). The grouping of the words was implemented before the main statistical analysis by a member of the research group (MG) who did not participate in the subsequent statistical analysis. This procedure, although subjective, was necessary since the independent participation of words with a small conceptual difference and large frequency differences would hide the influence of the construct described by them. The complete list of grouped words is presented in Additional file 1: Table S1.

### Statistical analysis

For each one of the eight categories, the frequency of all combinations between the first and the second word as well as between the second and the third word was counted, and the aggregated list of all word combinations was used to define the symmetric adjacency matrix from which the undirected graph of words was created. To quantify the importance of each word in the network, the authoritative [33], betweenness [34, 35], pagerank, and eigenvector [36, 37] centrality indexes were computed. The four centrality indexes were positively correlated (Table 1), indicating the similarity of their performance in representing the significance of the word in the corresponding graph. The authority index was selected as the one to participate in the subsequent analysis as the one that is easily interpreted having range between 0 and 1.

**Table 1:** Pearson correlation among the four centrality indexes

Centrality method	Authority	Betweenness	Pagerank
Betweenness	0.759**		
Pagerank	0.926**	0.865**	
Eigen	0.998**	0.752**	0.927**

**Correlation is significant at the 0.01 level (2-tailed)

For each one participant and each one of the eight-word categories, the average of the three word's authoritative centrality was computed as a single measure representing the overall centrality of the participant's word selection process.

Canonical correlation analysis was applied to identify significant associations between the eight mean word centralities and the set of the participant's psycho-demographic variables (age, gender, the nine psychopathology subscales, and the two shame subscales). Then, a path model was formulated to quantify the effects of the psycho-demographic variables on the word centralities indicated as significant by canonical correlation. The maximum likelihood method was used to estimate the parameters of the model.

All data were analyzed using SPSS statistical package (version 21) and R statistical language [38] equipped with lavaan, tidyverse, and tidygraph packages [39, 40].

## Results

In total, 626 words were recorded as responses to the eight different stimulus words (Additional file 1: Figures S1 and S2). The category of words related to nightlife had the most significant variation of different words (i.e., 93), while words concerning smoking had the most negligible variation (i.e., 64). The authority centrality index showed an extremely strong correlation with Pagerank and Eigen centrality indexes, having also a significant positive correlation with the remaining Betweenness index, thus chosen as an index representing the centrality of each word also has the advantage to be straightforwardly interpreted since its range is by definition from 0 to 1 (Table 1).

The distribution of the words according to their authoritative centrality index in the corresponding graph is presented in Table 2. The five words with the largest centrality and the corresponding cumulative frequency of appearance are presented in Additional file 1: Table S2.

**Table 2:** Word’s centrality distribution for each category

Centrality	Diet	Exercise	Smoking	Alcohol	Nightlife	Lifestyle	Disease	Health	Total
0.00–0.05	15	19	8	19	10	18	18	17	124
0.05–0.10	14	16	18	8	15	15	9	12	107
0.10–0.15	14	9	6	8	10	14	10	15	86
0.15–0.20	7	9	4	9	9	7	8	8	61
0.20–0.25	8	4	1	5	4	5	6	5	38
0.25–0.30	5	5	4	3	7	5	4	3	36
0.30–0.35	5	3	3	2	1	3	5	8	30
0.35–0.40	2	3	2	3	3	6	2	6	27
0.40–0.45	3	3	2	2	4	0	3	1	18
0.45–0.50	1	1	3	3	1	2	1	1	13
0.50–0.55	2	0	3	2	1	5	1	1	15
0.55–0.60	2	3	3	2	2	4	1	1	18
0.60–0.65	0	3	1	2	0	2	0	0	8
0.65–0.70	0	0	1	2	0	0	1	1	5
0.70–0.75	1	0	3	0	1	2	1	0	8
0.75–0.80	1	0	1	2	0	0	3	0	7
0.80–0.85	0	0	0	2	0	2	1	0	5
0.85–0.90	1	0	0	0	1	1	1	0	4
0.90–0.95	1	1	0	0	2	0	1	0	5
0.95–1.00	1	1	1	3	1	2	1	1	11
Total words	83	80	64	77	72	93	77	80	626
Mean	0.22	0.20	0.26	0.27	0.23	0.25	0.24	0.19	0.23

For each one participant and each one of the eight-word categories, the average of the three selected words authoritative centrality was computed as a single measure representing the overall centrality of the participant's word selection process.

Table 3 summarizes the psychological and centrality measures of the sample that participated in the canonical correlation analysis. The correlation coefficients among the variables of the two sets are presented in Additional file 1: Table S3.

**Table 3:** Descriptive statistics of the psychological and the centrality scores

	M (SD)	Skewness	Kurtosis
*Psychopathology*			
Somatization (SOM)	7.7 (6.9)	1.26	1.72
Obsessive compulsive (OC)	9.0 (6.7)	0.68	− 0.22
Interpersonal sensitivity (IS)	6.4 (5.4)	1.35	2.28
Depression (DEP)	10.2 (8.6)	1.17	1.28
Anxiety (ANX)	5.3 (5.9)	1.76	3.91
Hostility (HOS)	3.7 (4.2)	1.85	4.13
Phobic anxiety (PHB)	2.2 (3.6)	2.73	8.24
Paranoid ideation (PAR)	5.3 (4.3)	0.95	0.8
Psychotism (PSY)	4.9 (4.6)	1.63	3.41
General Symptom Index (GSI)	0.67 (0.5)	1.30	2.11
*Shame*			
External shame (OAS)	13.9 (10.0)	0.84	0.41
Internal shame (ESS)	45.3 (13.1)	0.74	0.26
*Participants centrality indexes*			
Diet (CTF)	0.58 (0.19)	− 0.46	− 0.12
Exercise (CTE)	0.52 (0.17)	− 0.60	0.62
Smoking (CTS)	0.58 (0.16)	− 0.43	− 0.08
Alcohol (CTA)	0.59 (0.19)	− 0.48	− 0.28
Nightlife (CTN)	0.63 (0.20)	− 0.66	− 0.05
Lifestyle (CTL)	0.53 (0.19)	− 0.49	0.09
Disease (CTD)	0.61 (0.21)	− 0.70	− 0.08
Health (CTH)	0.44 (0.19)	0.28	0.00

The tests of dimensionality for the canonical correlation analysis showed that three out of the eight canonical dimensions were statistically significant at the 0.05 level, explaining 40.4% of the variance between the two sets of variables (Table 4). Dimension 1 had a canonical correlation of 0.406, explaining 16.5% of the variance among the two sets of variables. For dimensions 2 and 3, the canonical correlations were 0.368 and 0.323, corresponding to 13.5% 10.4% of the explained variance among the two sets of variables.

**Table 4:** Canonical correlation and tests of canonical dimensions

Dimension	Canonical correlations	Percent of common variance explained (%)	Wilk's	Chi-SQ	*df*	*p*
1	0.406	16.5	0.510	169.850	104	0.000
2	0.368	13.5	0.610	124.468	84	0.003
3	0.323	10.4	0.706	87.857	66	0.037
4	0.287	8.2	0.788	60.117	50	0.155
5	0.258	6.7	0.859	38.412	36	0.361
6	0.217	4.7	0.920	20.985	24	0.640
7	0.161	2.6	0.966	8.823	14	0.842
8	0.093	0.9	0.991	2.204	6	0.900

The first canonical dimension indicated an effect of age (b_AGE_ = 0.723), interpersonal sensitivity (b_IS_ = 1.086), depression (b_DEP_ = − 0.713) and anxiety (b_ANX_ = 0.413) on the centrality of the words concerning nightlife (b_CTN_ = − 0.685), health (b_CTH_ = 0.443) and diet (b_CTF_ = − 0.495). The second one, indicated an effect of gender (b_GND_ = − 0.629), depression (b_DEP_ = − 0.652) and shame (b_OAS_ = − 0.612, b_ESS_ = 0.571) on the centrality of the words concerning lifestyle (b_CTL_ = 0.630), alcohol consumption (b_CTA_ = − 0.440) and health (b_CTH_ = 0.415), while the third dimension indicated a relation between hostility (b_HOS_ = 0.687), depression (b_DEP_ = -0.555) and external shame (b_OAS_ = 0.412) and the centralities of the alcohol consumption (b_CTA_ = − 0.771), diet (b_CTF_ = − 0.422) and nightlife (b_CTN_ = 0.381) categories (Table 4). Thus, somatization, obsessive compulsive, phobic anxiety, paranoid ideation and psychotism emerged to be the least important psychological characteristics concerning their affection on the centrality of the associated words, while on the other hand, the words concerning exercise, smoking, and disease were the least affected words. The standardized canonical coefficients for the first three dimensions across both sets of variables are presented in Table 5.

**Table 5:** Loadings and standardized coefficients of the three significant dimensions

	Dimension 1		Dimension 2		Dimension 3	
	Loadings	Std. Coeff	Loadings	Std. Coeff	Loadings	Std. Coeff
*Word category*						
Diet (CTF)	− 0.495	− 0.380	0.148	− 0.054	− 0.474	− 0.422
Exercise (CTE)	− 0.219	− 0.241	0.288	0.191	− 0.359	− 0.059
Smoking (CTS)	0.039	0.172	0.001	− 0.094	− 0.303	− 0.090
Alcohol (CTA)	− 0.113	− 0.062	− 0.157	− 0.440	− 0.798	− 0.771
Nightlife (CTN)	− 0.639	− 0.685	0.416	0.359	0.023	0.381
Lifestyle (CTL)	− 0.037	0.054	0.743	0.630	− 0.326	− 0.172
Disease (CTD)	0.288	0.360	0.295	0.132	− 0.350	− 0.180
Health (CTH)	0.443	0.464	0.547	0.415	− 0.226	− 0.035
*Psycho-demographics*						
Age (AGE)	0.578	0.723	− 0.151	− 0.052	− 0.531	− 0.373
Gender (GND)	0.152	0.039	− 0.717	− 0.629	0.077	0.074
Somatization (SOM)	0.178	− 0.315	0.374	0.313	0.199	− 0.028
Obs. compulsive (OC)	0.200	− 0.152	0.351	0.223	0.210	− 0.016
Inter. sensitivity (IS)	0.498	1.086	0.301	0.505	0.223	− 0.384
Depression (DEP)	0.239	− 0.713	0.234	− 0.652	0.156	− 0.555
Anxiety (ANX)	0.316	0.413	0.318	0.178	0.394	0.087
Hostility (HOS)	0.353	0.369	0.245	0.221	0.749	0.687
Phobic anxiety (PHB)	0.403	− 0.108	0.118	0.040	0.382	0.256
Par. ideation (PAR)	0.345	− 0.067	0.187	− 0.013	0.341	0.219
Psychotism (PSY)	0.418	0.311	0.117	− 0.433	0.434	0.108
External shame (OAS)	0.102	− 0.211	− 0.064	− 0.612	0.500	0.412
Internal shame (ESS)	0.048	− 0.229	0.430	0.571	0.260	0.013

**Fig. 1:**
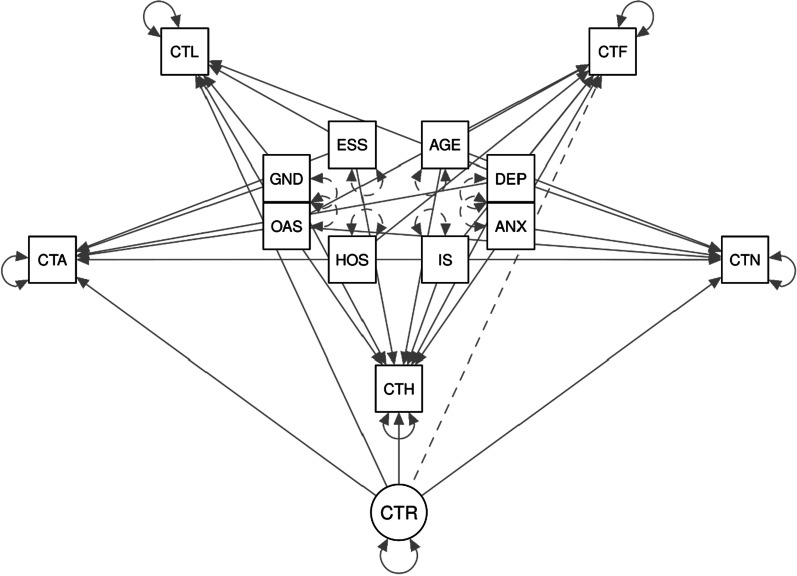
Measurement model (CTF ~ AGE + DEP + ANX + IS + HOS + OAS, CTN ~ AGE + DEP + ANX + IS + HOS + OAS + ESS, CTH ~ AGE + DEP + ANX + IS + GND + OAS + ESS, CTA ~ GND + DEP + OAS + HOS + ESS, CTL ~ GND + DEP + OAS + ESS, CTR =  ~ CTF + CTN + CTH + CTA + CTL). *Notes*: CTF, diet; DEP, depression; ANX, anxiety; IS, interpersonal sensitivity; HOS, hostility; OAS, external shame; CTN, nightlife; ESS, internal shame; CTH, health; GND, gender; CTA, alcohol; CTL, lifestyle; CTR, representations

From the canonical correlation analysis results, the model of Fig. 1 was formulated aiming further to explore the psychopathology and sociodemographic effect on word centralities. The core assumption of the tested model is that the five-word categories consist of one latent variable reflecting the subject's overall word centrality. Further, the model aims to enlighten the significance of the direct effects of the psycho-demographic variables on word's centrality as suggested by the canonical correlation analysis.

Comparative fit index (CFI) 0.992, Tucker Lewis index (TLI) 0.975, goodness-of-fit index (GFI) 0.976, Normed fit index (NFI) 0.897, standardized root mean square residual (SRMR) 0.022, and root mean square error of approximation (RMSEA) 0.015 (95% CI 0–0.060) suggested that the model showed an acceptable fit to the data. The standardized regression coefficients are presented in Table 6.

**Table 6:** Path model's parameters

	Estimate	95% CI		SE	z value	*p*	Std. lv^*^	Std. all^**^	R^2^
		Lower	Upper						
*CTR*									
CTF	1.000	1.000	1.000				0.043	0.228	
CTN	2.332	0.342	4.322	1.015	2.297	0.022	0.101	0.513	
CTH	0.986	− 0.026	1.998	0.516	1.910	0.056	0.043	0.233	
CTA	1.925	0.282	3.567	0.838	2.297	0.022	0.084	0.424	
CTL	1.67	0.219	3.121	0.740	2.256	0.024	0.072	0.382	
*CTF*									0.116
AGE	− 0.015	− 0.033	0.003	0.009	− 1.657	0.097	− 0.015	− 0.101	
**DEP**	**0.008**	**0.002**	**0.013**	**0.003**	**2.802**	**0.005**	**0.008**	**0.345**	
ANX	− 0.001	− 0.007	0.006	0.003	− 0.234	0.815	− 0.001	− 0.024	
**IS**	− **0.010**	− **0.017**	− **0.002**	**0.004**	− **2.479**	**0.013**	− **0.010**	− **0.268**	
**HOS**	− **0.009**	− **0.016**	− **0.002**	**0.003**	− **2.571**	**0.010**	− **0.009**	− **0.196**	
OAS	− 0.006	− 0.034	0.022	0.014	− 0.421	0.674	− 0.006	− 0.032	
*CTN*									0.255
**AGE**	− **0.038**	− **0**.**056**	− **0**.**021**	**0**.**009**	− **4**.**266**	**< 0.001**	− **0.038**	− **0.247**	
DEP	0.004	− 0.001	0.01	0.003	1.612	0.107	0.004	0.191	
ANX	− 0.002	− 0.008	0.005	0.003	− 0.535	0.593	− 0.002	− 0.053	
IS	− 0.007	− 0.015	0	0.004	− 1.963	0.050	− 0.007	− 0.203	
HOS	− 0.005	− 0.011	0.002	0.003	− 1.391	0.164	− 0.005	− 0.102	
OAS	− 0.012	− 0.042	0.019	0.016	− 0.739	0.460	− 0.012	− 0.06	
**ESS**	**0.028**	**0.007**	**0.05**	**0.011**	**2.601**	**0.009**	**0.028**	**0.194**	
*CTH*									0.136
AGE	0.014	− 0.003	0.031	0.009	1.644	0.100	0.014	0.098	
**DEP**	− **0.007**	− **0.013**	− **0.002**	**0.003**	− **2.777**	**0.005**	− **0.007**	− **0.346**	
ANX	0.005	− 0.001	0.011	0.003	1.56	0.119	0.005	0.154	
**IS**	**0.011**	**0.004**	**0.019**	**0.004**	**3.09**	**0.002**	**0.011**	**0.334**	
GND	− 0.039	− 0.085	0.007	0.024	− 1.653	0.098	− 0.039	− 0.103	
**OAS**	− **0.046**	− **0.075**	− **0.017**	**0.015**	− **3.113**	**0.002**	− **0.046**	− **0.255**	
ESS	0.014	− 0.007	0.034	0.01	1.305	0.192	0.014	0.099	
*CTA*									0.247
GND	0.026	− 0.022	0.075	0.025	1.064	0.287	0.026	0.065	
DEP	0.003	− 0.001	0.006	0.002	1.401	0.161	0.003	0.117	
OAS	− 0.021	− 0.052	0.009	0.015	− 1.392	0.164	− 0.021	− 0.112	
**HOS**	− **0.011**	− **0.018**	− **0.005**	**0.003**	− **3.469**	**0.001**	− **0.011**	− **0.245**	
ESS	− 0.001	− 0.022	0.021	0.011	− 0.057	0.955	− 0.001	− 0.004	
*CTL*									0.207
**GND**	− **0.065**	− **0.112**	− **0.019**	**0.024**	− **2.734**	**0.006**	− **0.065**	− **0.168**	
DEP	0.002	− 0.001	0.005	0.002	1.175	0.240	0.002	0.09	
**OAS**	− **0.035**	− **0.064**	− **0.006**	**0.015**	− **2.338**	**0.019**	− **0.035**	− **0.187**	
ESS	0.016	− 0.005	0.037	0.011	1.492	0.136	0.016	0.113	

Statistically significant paths are highlighted in bold

c^2^(50) = 164,1, *p* < .001; RMSEA = 0.015, CFI = 0.992, TLI = 0.975

CTF, diet; DEP, depression; ANX, anxiety; IS, interpersonal sensitivity; HOS, hostility; OAS, external shame; CTN, nightlife; ESS, internal shame; CTH, health; GND, gender; CTA, alcohol; CTL, lifestyle; CTR, representations

^*^Dependent variables are standardized

^**^Completely standardized solution

The model explained the 11.6% of the diet words centrality, 25.5% of nightlife, 13.6% of health, 24.7% of alcoholic beverage, and 20.7% of the lifestyle category. The centrality of the words concerning health was not significantly regressed on the latent centrality variable (*p* = 0.056), being, however, marginally rejected as a regressor. On the other hand, the latent centrality variable was significantly determined by the other four partial centralities that participated in the model.

Older respondents found to give more decentralized words when “Nightlife” was the stimulus word (b_std_ = − 0.247, b = − 0.038, 95% CI =  − 0.056 to − 0.021, *p* ≤ 0.001), while women provided more centrally positioned words than men in the lifestyle words graph (b_std_ = − 0.168, b = − 0.065, 95% CI − 0.112 to − 0.019, *p* = 0.006).

Depression (DEP) had a significant positive effect on the centrality of words concerning diet (b_std_ = 0.345, b = 0.008, 95% CI 0.002–0.013, *p* = 0.005) and a significantly negative effect on health words centralities (b_std_ = − 0.346, b = − 0.007, 95% CI − 0.013 to − 0.002, *p* = 0.005), a finding showing that subjects with increased levels of depression tend to choose more centrally positioned words when the stimulus word is diet, and give more decentralized responses when the stimulus word is health.

External shame (OAS) had a significant negative effect on word centrality when the stimulus word is health (b_std_ = − 0.255, b = − 0.046, 95% CI − 0.075 to − 0.017, *p* = 0.002) or lifestyle (b_std_ = − 0.187, b = − 0.035, 95% CI − 0.064 to − 0.006, *p* = 0.019), a finding reflecting that higher external shame corresponds to more decentralized words to the corresponding graphs for these two categories. On the other hand, higher internal shame (ESS) found to be associated with word choice with greater centrality when nightlife was the stimulus word (b_std_ = 0.194, b = 0.028, 95% CI 0.007–0.05, *p* = 0.009).

Hostility subscale found to have a significant negative effect on alcohol consumption (b_std_ = − 0.245, b = − 0.011, 95% CI − 0.018 to − 0.005, *p* = 0.001) and diet word categories (b_std_ = − 0.196, b = − 0.009, 95% CI − 0.016 to − 0.002, *p* = 0.01), a finding that reflects the tendency of a subject with higher score in hostility subscale to supply words positioned less centrally in the corresponding words graph. Finally, subjects with higher interpersonal sensitivity were found to supply more decentralized words when the stimulus word was diet (b_std_ = − 0.268, b = − 0.010, 95% CI − 0.017 to − 0.002, *p* = 0.013) and more centrally positioned words concerning health (b_std_ = 0.334, b = 0.011, 95% CI 0.004–0.019, *p* = 0.002).

## Discussion

This study investigated whether psychological deviations correspond to deviations in the centrality of the associated words selected to describe eight social representations of health and lifestyle. By visualizing a graph theory approach [41], this study highlights the interconnection of social representations with psychopathological symptoms, demonstrating differences in the extent to which a representation is commonly accepted and rooted in words. More importantly, the graph representation of free word association can emerge as a method allowing for detecting such word variations. Overall, a not homogeneous effect of a psycho-demographic variable on word's centrality among the eight-word categories was established, suggesting a different degree of social representation agreement concerning those concepts' meanings. Notably, it was found that the word categories of exercise, smoking, and disease were the least influenced categories by the psycho-demographic variables showing that the social representations of those concepts may have a more uniform semantic distribution in the word usage. Thus, Greek society as an indicative western world society appears to be homogenized in terms of representations related to exercise, smoking, and the impact of the disease on human life. This finding may be attributed to the society's exposure to a broad and long-standing unambiguous description of these concepts, a practice that has inevitably led the society to the acceptance of a common meaning for those representations following the concept's shared meanings or shared conceptual maps [42].

In the context of the exploratory structural model that was tested, the centralities of the categories concerning diet, alcoholic beverage consumption, nightlife, and lifestyle was founded to consist of the same latent variable, suggesting that there is a similar effect of age, gender, and psychopathology on the centralities of the words. These findings may reveal that these social representations have a comparable level of word homogenization, nevertheless offering the possibility of personalized expression. For example, in a study of climate change, the authors found a common core set of concepts, but there were also many differences in how climate change is framed and conceived by respondents [43]. The centrality of the words concerning health was founded to be marginally non-significantly loaded on that latent factor showing that social representations about health are affected by the psycho-demographic variables differently than the other four-word groups. This result ties nicely with previous studies wherein sociodemographic variables such as age, gender, education, and socio-economic status, should be taken into account in the study of social representations [3, 11].

Also, differences were reported concerning the effect of age, gender, psychopathology, and shame on the centralities of the five-word categories providing insight into how those factors are reflected in the differences of the social representations within the same Greek cultural context. Older respondents were found to give more decentralized words when "Nightlife" was the stimulus word, a finding reflecting the different pace at which people move away from nightlife experiences as they are getting older. The finding that women provided more centrally positioned words than men in the lifestyle words graph reflects the known fact that women have healthier dietary habits, a higher rate of physical activity, and a lower rate of smoking and obesity than men [44]. A healthy dietary pattern previously reported relates to decreasing the risk of depression [45]. Thus one would assume that a similar effect would appear between depression and the centrality of the words selected to describe the social representation of diet. The present study's findings seem to provide hints towards a different direction since subjects with increased levels of depression were found to choose more centrally positioned words in the diet, giving more decentralized responses when the stimulus word was health. Those findings suggest a higher complexity pattern of the social representations concerning diet and health and the relation with depression disorder and a possible interaction effect with other factors that remain to be demonstrated in future studies.

As reflected in the external shame score, higher feelings of inferiority correspond to more decentralized words to the network graph of lifestyle words category, indicating higher subject's desire to manifest an identity with stronger personal characteristics. The analogous effect of external shame on the centrality of the words concerning health is not possible to visibly interpreted, suggesting a possible interaction with physical health problems. This factor was not met in our study. The negative feelings associated with what one thinks and feels about oneself are reflected in the internal shame score. Internal shame score was found to significantly affect the centrality of the words concerning the social representation of nightlife, showing greater subject's insecurity to move away from the social norms describing human activities of entertainment of that kind. These findings support the notion that shame should be considered as a determinant of health [46]. The link between extensive alcohol consumption and hostility has been well documented in the literature [47, 48], while analogous results also indicate the relation of hostility with eating disorders [49]. The relations as mentioned above are confirmed as far as the way of expression is concerned since hostility score found to have a significant negative effect on alcohol consumption and diet word categories. This finding reflects the tendency of a subject with a higher hostility score to stay away from social norms and avoid using mainstream words to describe those representations providing words positioned less centrally in the word graphs of alcohol and diet. Interpersonal sensitivity has been previously reported to be connected with eating disorder symptoms [50], being also related to higher general morbidity and mortality [51]. Individuals with higher interpersonal sensitivity were found to provide more decentralized words when the stimulus word was diet indicating a greater distancing from social norms. At the same time, more considerable interpersonal sensitivity was connected to more centrally positioned words concerning health, demonstrating a common-sense agreement on health representation.

### Limitations and further remarks

The literature review did not provide any previous research on free associations following the analysis method of the present study. Thus, it is essential to describe all the sources of ambiguity with respect to our method of analysis to allow the reader to evaluate the study's findings correctly.

Firstly, the concept of the importance of a node in a graph is ill-defined, and that is the reason that many centrality measures have been proposed in the graph theory. The four centrality measures that were computed in the present article are commonly reported in the literature. They found to be highly correlated, showing that in the context of the present study, centrality is reliably demonstrates each word’s significance in the corresponding graph. Among the four selected centrality indexes, the authoritative centrality index was prefered to be used  in the subsequent analysis due to the ease of interpretation. It is not known whether there will be significant differences in the reported findings under a different centrality index choice among the other theoretically available indexes.

Concerning the structural equation model, the baseline model's RMSEA fit index was equal to 0,093, smaller than 0,158, a limit below which the fit indexes are reported to be not very informative [52], a finding that possibly reflects the overall small correlation among the eight centralities and the psychometric variables (Additional file 1: Table S3). Additionally, a simple direct effect model was chosen to test the effects of the psycho-demographic variables on word centralities, a non-optimal option since there is strong evidence in the literature that there are significant effects of age and gender on various social groups on psychopathology [53]. Lastly, various interactions have been suggested between gender, psychopathology, and human habits that were investigated in the present study [54]. These facts may suggest a richer path model where psychopathology and shame would also regress on age and gender could be more realistic. However, a model of greater complexity did not acceptably fit our data, suggesting future analogous research to a larger sample.

## Conclusion

Notwithstanding these shortcomings, the results of this study can give valuable contributions to the construction of social representations about health, illness, and lifestyle behaviours with possible application in the field of health marketing. Studying how the centrality of a word interacts with the psychopathological state in the general population within the same group of individuals may help researchers objectify "new" scientific and emotional issues associated with the nature and meaning of such representations. Our study found differences among the central core words and categories provided by individuals with different levels of psychopathology, indicating that the dynamics of social representations are subject to socio-cultural and individual emotional phenomena. Further work is required to examine whether the reflections of psychopathology on social representations are casual, making also possible an early identification of psychopathological process through the word usage within a group of individuals.

## Supplementary Information

**Additional file 1. Figure S1**. Word graphs for diet, exercise, alcoholic beverages and nightlife (up to down and left to right).** Table S1**. Words that grouped together.** Table S2**. The 5 words with the largest centrality in each word category.** Table S3**. Pearson correlation among the eight centralities, the demographics, and the psychopathology scales. Free association task questionnaire.

## Data Availability

The datasets generated and/or analysed during the current study are not publicly available due to the Greek law regarding the type of data including grounds of confidentiality and anonymity but are available from the corresponding author on reasonable request.

## Abbreviations

ANX: Anxiety
b_std_: Standardized regression coefficient
CFI: Comparative Fit Index
CI: Confidence interval
CTA: Alcohol authority centrality
CTD: Disease authority centrality
CTE: Exercise authority centrality
CTF: Diet authority centrality
CTH: Health authority centrality
CTL: Lifestyle authority centrality
CTN: Nightlife authority centrality
CTS: Smoking authority centrality
DEP: Depression
ESS: Internal shame
GFI: Goodness-of-Fit Index
GSI: General Symptom Index
HOS: Hostility
IS: Interpersonal sensitivity
NFI: Normed Fit Index
OAS: External shame
OC: Obsessive compulsive
PAR: Paranoid ideation
PHB: Phobic anxiety
PSY: Psychotism
RMSEA: Root mean square error of approximation
SCL-90-R: Symptom check list 90-revised
SOM: Somatization
SRMR: Standardized root mean square residual
TLI: Tucker Lewis index
