# Supplementary material for: The effects of psychopathology and shame on social representations of health and lifestyle behaviours via free association: a graph analysis approach
Source: BMC Psychol. 2021 Oct 29;9:168. doi: 10.1186/s40359-021-00671-x (PMC8555264; doi:10.1186/s40359-021-00671-x)
Supplement: Supplementary file 1 — Additional file 1. Figure S1. Word graphs for diet, exercise, alcoholic beverages and nightlife (up to down and left to right). Table S1. Words that grouped together. Table S2. The 5 words with the largest centrality in each word category. Table S3. Pearson correlation among the eight centralities, the demographics, and the psychopathology scales. Free association task questionnaire. [file 40359_2021_671_MOESM1_ESM.docx]

**Additional file 1**

The effects of psychopathology and shame on social representations of health and lifestyle behaviours via free association: A graph analysis approach.

Evangelia Briseniou PhD, Nikolaos Skenteris MD, PhD, Chryssi Hatzoglou MD, PhD, GeorgeTsitsas PhD, Epaminondas Diamantopoulos PhD Elena Dragioti PhD Mary Gouva PhD

**Supplementary Information**

Contents

Figure S1: Word graphs for diet, exercise, alcoholic beverages and nightlife (up to down and left to right)……………………………………………………3

[Figure S2: Word graphs for smoking, lifestyle, disease and health (up to down and left to right). 4](#_Toc61354600)

[Table S1: Words that grouped together 5](#_Toc61354601)

[Table S2: The 5 words with the largest centrality in each word category 7](#_Toc61354602)

[Table S3: Pearson correlation among the eight centralities, the demographics and the psychopathology scales. 9](#_Toc61354603)

[Free association task questionnaire 11](#_Toc61354604)


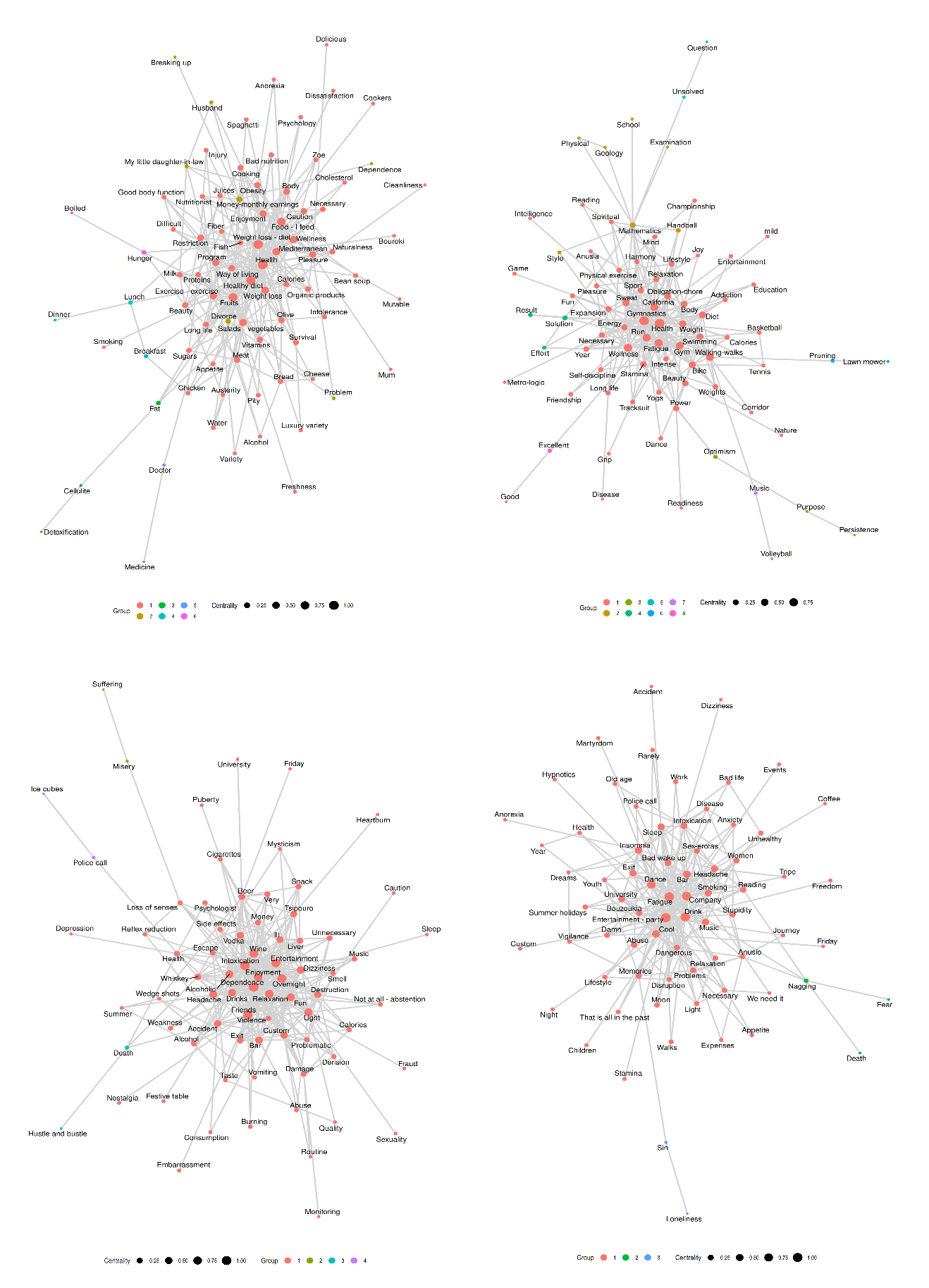
 Figure S1: Word graphs for diet, exercise, alcoholic beverages, and nightlife (up to down and left to right)

#
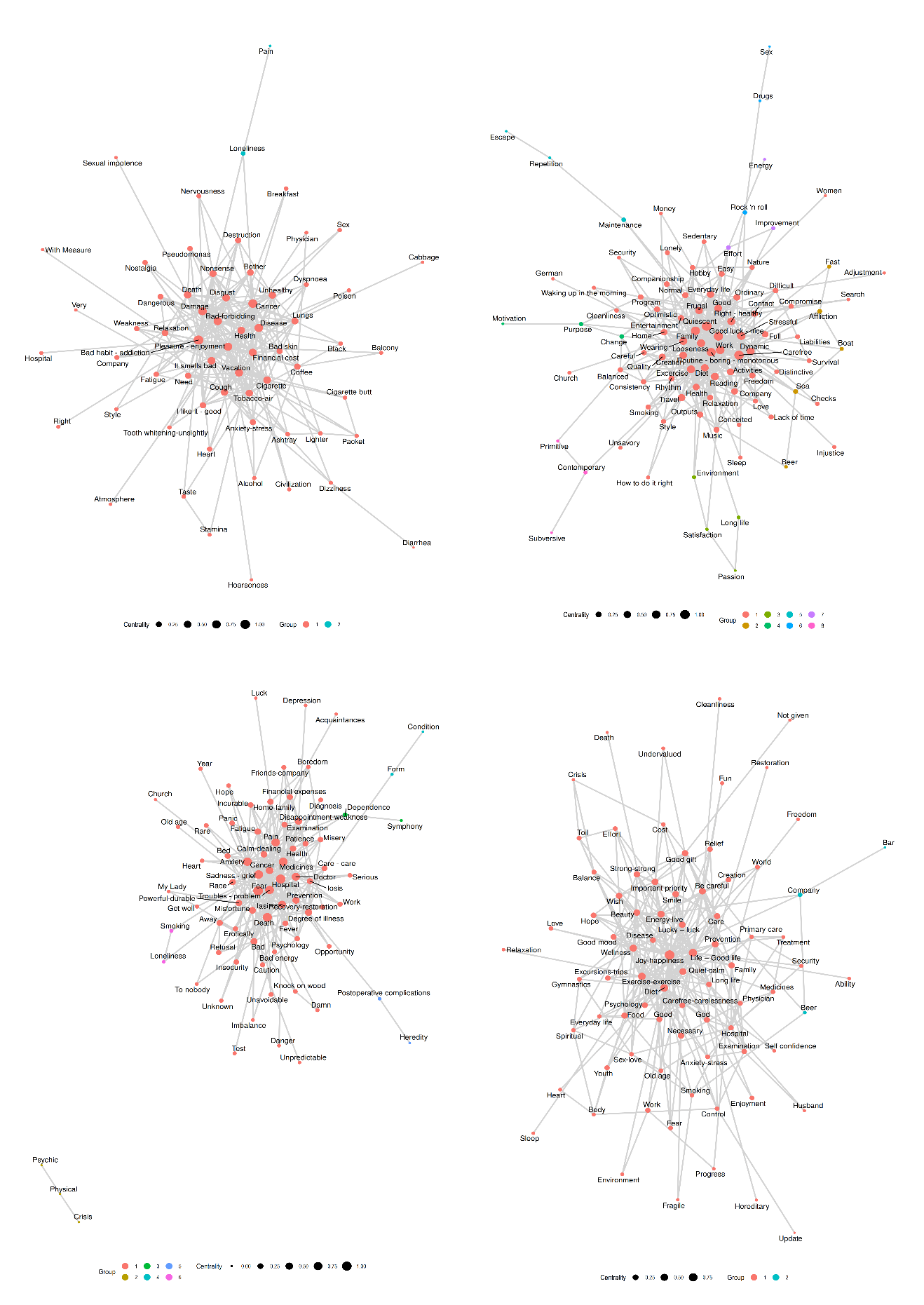
 Figure S2: Word graphs for smoking, lifestyle, disease, and health (up to down and left to right).

| Table S1: Words that grouped together | | | |
| --- | --- | --- | --- |
| Diet | Exercise | Smoking | Alcoholic beverage |
| Gymnastics - Exercise | Walking-Saunter | Bad habit - Addiction | Fun - Entertainment |
| Salads - Vegetables | Obligation – Tedious task | I like it - Good | In moderation - A little |
| Food - Satiate | Moderation-Logic | Bad - Prohibitive | Friends - Company |
| Money-Monthly Earnings |  | Pleasure - Enjoyment | Not at all - Abstention |
| Weight Loss - Diet |  | Smoke - Air |  |
| My little daughter-Children |  | Anxiety - Stress |  |
| Luxury-Variety |  |  |  |
| Nightlife | Lifestyle | Disease | Health |
| Friends - Company | Routine - Boring | Anxiety – Agony | Be careful - Careful |
| Fun - Have fun - Entertainment - Feast | Have a good time - Nice | Sadness - Distress | Important - Priority |
| Cool beans – A lot of fun | Difficult - Bad | Suffering - Problem | Strength - Ηeartiness |
| Stupid - Pointless | Proper – Healthy | Home - Family | Life - Good life |
| Fatigue - Suffering | Carefree – Light-hearted | Aid- Care | Fortune - Lucky |
| Sex-Love | Regular - Normal | Coolness – Treatment | Carefree – Light-hearted |
| In moderation - A little |  | Strength - Endurance | Sports - Exercise |
| Summer-Holidays |  | Disability - Weakness | Tranquillity - Calm |
|  |  | Recovery - Rehabilitation | Energy - Happiness |
|  |  | Friends - Company | Joy-Happiness |
|  |  |  | Good-Gift |
|  |  |  | Sex-Love |
|  |  |  | Excursions - Travels |
|  |  |  | Anxiety - Stress |

| Table S2: The 5 words with the largest centrality in each word category | | |
| --- | --- | --- |
|  | Diet | Exercise |
| 1 | Health, N ^(1)^= 119, c = 1 | Health, N = 104, c = 1 |
| 2 | Weakness - Diet, N = 78, c = 0,94 | Gymnastics, N = 80, c = 0,93 |
| 3 | Healthy Diet, N = 72, c = 0,86 | Wellness, N = 45, c = 0,65 |
| 4 | Fruits, N = 68, c = 0,78 | Fatigue, N = 42, c = 0,65 |
| 5 | Food - Satiate, N = 47, c = 0,72 | Running, N = 61, c = 0,64 |
|  | Nighlife | Lifestyle |
| 1 | Fun - Entertainment, N = 119, c = 1 | Quiescent, N = 68, c = 1 |
| 2 | Fatigue - Suffering, N = 86, c = 0,94 | Carefree - Light-hearted, N = 27, c = 0,95 |
| 3 | Drink, N = 106, c = 0,93 | Have a good time - Nice, N = 29, c = 0,87 |
| 4 | Friends - Company, N = 96, c = 0,86 | Family, N = 43, c = 0,83 |
| 5 | Dance, N = 33, c = 0,71 | Routine - Boring, Ν =44, c = 0,82 |
|  | Smoking | Alcoholic Beverage |
| 1 | Bad habit - Addiction, N = 121, c = 1 | Dependence, N = 58, c = 1 |
| 2 | Cancer, N = 74, c = 0,80 | Intoxication, N = 58, c = 0,97 |
| 3 | Sickness, N = 51, c = 0,74 | Friends - Company, N = 60, c = 0,95 |
| 4 | Pleasure - Enjoyment, N = 51, c = 0,71 | Nightlife, N = 47, c = 0,84 |
| 5 | Bad - Prohibitive, N = 48, c = 0,70 | Fun - Entertainment, N = 58, c = 0,81 |
|  | Disease | Health |
| 1 | Fear, N = 69, c = 0,1 | Joy - Happiness, N = 128, c = 1 |
| 2 | Hospital, N = 87, c =0,95 | Life - Good life, N = 59, c = 0,66 |
| 3 | Death, N = 50, c = 0,86 | Diet, N = 44, c = 0,58 |
| 4 | Sadness - Distress, N = 28, c = 0,81 | Sports - Exercise, N = 41, c = 0,52 |
| 5 | Anxiety – Agony, N = 31, c = 0,79 | Wellness, N = 38, c = 0,48 |
| (1) Count of appearances in all three positions | | |

| Table S3: Pearson correlation among the eight centralities, the demographics, and the psychopathology scales. | | | | | | | | | | | | | | | | | | | | |
| --- | --- | --- | --- | --- | --- | --- | --- | --- | --- | --- | --- | --- | --- | --- | --- | --- | --- | --- | --- | --- |
|  | CTF | CTE | CTS | CTA | CTN | CTL | CTD | CTH | AGE | GND | SOM | OC | IS | DEP | ANX | HOS | PHB | PAR | PSY | OAS |
| CTE | .263^**^ |  |  |  |  |  |  |  |  |  |  |  |  |  |  |  |  |  |  |  |
| CTS | .183^**^ | .106 |  |  |  |  |  |  |  |  |  |  |  |  |  |  |  |  |  |  |
| CTA | .064 | .161^**^ | .134* |  |  |  |  |  |  |  |  |  |  |  |  |  |  |  |  |  |
| CTN | .170^**^ | .029 | .139^*^ | .250^**^ |  |  |  |  |  |  |  |  |  |  |  |  |  |  |  |  |
| CTL | .196^**^ | .123^*^ | .175^**^ | .135^*^ | .200^**^ |  |  |  |  |  |  |  |  |  |  |  |  |  |  |  |
| CTD | .037 | .112 | .157^**^ | .208^**^ | .197^**^ | .116 |  |  |  |  |  |  |  |  |  |  |  |  |  |  |
| CTH | .032 | .210^**^ | -.017 | .158^**^ | .065 | .184^**^ | .202^**^ |  |  |  |  |  |  |  |  |  |  |  |  |  |
| AGE | -.066 | -0.04 | .064 | .095 | -.155^**^ | .031 | 0.10 | .085 |  |  |  |  |  |  |  |  |  |  |  |  |
| GND | -.120^*^ | -.097 | -.016 | .030 | -.085 | -.206^**^ | -.078 | -.085 | .064 |  |  |  |  |  |  |  |  |  |  |  |
| SOM | .016 | -.094 | -.025 | -.101 | -.011 | .102 | .066 | .026 | .384^**^ | -.238^**^ |  |  |  |  |  |  |  |  |  |  |
| OC | .008 | -.067 | -.019 | -.105 | -.005 | .076 | .101 | .045 | .213^**^ | -.237^**^ | .702^**^ |  |  |  |  |  |  |  |  |  |
| IS | -.084 | -.051 | -.008 | -.095 | -.070 | .046 | .188^**^ | .062 | .202^**^ | -.162^**^ | .653^**^ | .767^**^ |  |  |  |  |  |  |  |  |
| DEP | -.002 | -.085 | .025 | -.089 | -.022 | .073 | .144* | -.016 | .263** | -.256** | .743** | .830** | .824** |  |  |  |  |  |  |  |
| ANX | -.034 | -.094 | .042 | -.151** | -.023 | .083 | .052 | .042 | .237** | -.182** | .755** | .753** | .725** | .799** |  |  |  |  |  |  |
| HOS | -.139* | -.103 | -.09 | -.226** | -.069 | -.013 | .031 | .024 | .014 | -.032 | .451** | .497** | .521** | .524** | .578** |  |  |  |  |  |
| PHB | -.098 | -.09 | .036 | -.142* | -.091 | .048 | .061 | -.006 | .284** | -.085 | .611** | .641** | .698** | .681** | .740** | .399** |  |  |  |  |
| PAR | -.096 | -.068 | -.04 | -.122* | -.089 | .043 | .074 | -.004 | .181** | -.146** | .573** | .660** | .757** | .721** | .639** | .560** | .504** |  |  |  |
| PSY | -.036 | -.115* | .074 | -.176** | -.081 | -.016 | .068 | .041 | .174** | -.115** | .614** | .712** | .752** | .752** | .775** | .537** | .698** | .654** |  |  |
| OAS | -.069 | -.112 | -.028 | -.139* | .000 | -.059 | .053 | -.103 | .120** | -.042 | .434** | .529** | .591** | .553** | .565** | .432** | .464** | .520** | .573** |  |
| ESS | .003 | -.048 | .032 | -.089 | .098 | .067 | .132* | .041 | .101* | -.144** | .352** | .517** | .498** | .490** | .520** | .319** | .421** | .365** | .493** | .520** |
| **. Correlation is significant at the 0.01 level (2-tailed). | | | | | | | | | | | | | | | | | | | | |
| *. Correlation is significant at the 0.05 level (2-tailed).  Notes: CTE=Exercise authority centrality, CTF=Diet authority centrality, CTS=Smoking authority centrality, CTA=Alcohol authority centrality, CTN=Nightlife authority centrality, CTL=Lifestyle authority centrality, CTD=Disease authority centrality, CTH=Health authority centrality, AGE=Age, GND=Gender, SOM=Somatization, OC=Obsessive Compulsive, IS=Interpersonal Sensitivity, DEP= Depression, ANX= Anxiety, HOS= Hostility, PHB=Phobic Anxiety, PAR=Paranoid Ideation, PSY=Psychotism, OAS= External Shame, ESS= Internal Shame. | | | | | | | | | | | | | | | | | | | | |

# Free association task questionnaire

1.WHEN YOU HEAR TO THE WORD **DIET**, WHAT ARE THE FIRST THREE WORDS THAT COME TO YOUR MIND AND DESCRIBE IT?

| **Α)** | **Β)** | **C)** |
| --- | --- | --- |

2.WHEN YOU HEAR TO THE WORD **EXERCISE**, WHAT ARE THE FIRST THREE WORDS THAT COME TO YOUR MIND AND DESCRIBE IT?

| **Α)** | **Β)** | **C)** |
| --- | --- | --- |

3.WHEN YOU HEAR TO THE WORD **SMOKING**, WHAT ARE THE FIRST THREE WORDS THAT COME TO YOUR MIND AND DESCRIBE IT?

| **Α)** | **Β)** | **C)** |
| --- | --- | --- |

4.WHEN YOU HEAR TO THE WORD **ALCOHOLIC BEVERAGE**, WHAT ARE THE FIRST THREE WORDS THAT COME TO YOUR MIND AND DESCRIBE IT?

| **Α)** | **Β)** | **C)** |
| --- | --- | --- |

5. WHEN YOU HEAR TO THE WORD **NIGHTLIFE**, WHAT ARE THE FIRST THREE WORDS THAT COME TO YOUR MIND AND DESCRIBE IT?

| **Α)** | **Β)** | **C)** |
| --- | --- | --- |

6. WHEN YOU HEAR TO THE WORD **LIFESTYLE**, WHAT ARE THE FIRST THREE WORDS THAT COME TO YOUR MIND AND DESCRIBE IT?

| **Α)** | **Β)** | **C)** |
| --- | --- | --- |

7. WHEN YOU HEAR TO THE WORD **DISEASE**, WHAT ARE THE FIRST THREE WORDS THAT COME TO YOUR MIND AND DESCRIBE IT?

| **Α)** | **Β)** | **C)** |
| --- | --- | --- |

8. WHEN YOU HEAR TO THE WORD **HEALTH**, WHAT ARE THE FIRST THREE WORDS THAT COME TO YOUR MIND AND DESCRIBE IT?

| **Α)** | **Β)** | **C)** |
| --- | --- | --- |
